# Prevalence of potential drug-drug interactions and associated factors among outpatients and inpatients in Ethiopian hospitals: a systematic review and meta-analysis of observational studies

**DOI:** 10.1186/s40360-020-00441-2

**Published:** 2020-08-24

**Authors:** Wondim Ayenew, Getahun Asmamaw, Arebu Issa

**Affiliations:** 1 Department of Pharmaceutics, College of Health Science, School of Pharmacy, University of Gondar, Gondar, Ethiopia; 2 Department of Pharmacy, College of Health Science, Arba Minch University, Arba Minch, Ethiopia; 3 Department of Pharmaceutics and Social Pharmacy, College of Health Science, School of Pharmacy, Addis Ababa University, Addis Ababa, Ethiopia

**Keywords:** Drug-drug interactions, Hospitals, Ethiopia

## Abstract

**Background:**

Drug-drug interaction is an emerging threat to public health. Currently, there is an increase in comorbid disease, polypharmacy, and hospitalization in Ethiopia. Thus, the possibility of drug-drug interaction occurrence is high in hospitals. This study aims to summarize the prevalence of potential drug-drug interactions and associated factors in Ethiopian hospitals.

**Methods:**

A literature search was performed by accessing legitimate databases in PubMed/MEDLINE, Google Scholar, and Research Gate for English-language publications. To fetch further related topics advanced search was also applied in Science Direct and HINARI databases. The search was conducted on August 3 to 25, 2019. All published articles available online until the day of data collection were considered. Outcome measures were analyzed with Open Meta Analyst and CMA version statistical software. Der Simonian and Laird’s random effect model, I^2^ statistics, and Logit event rate were also performed.

**Results:**

A total of 14 studies remained eligible for inclusion in systematic review and meta-analysis. From the included studies, around 8717 potential drug-drug interactions were found in 3259 peoples out of 5761 patients. The prevalence of patients with potential drug-drug interactions in Ethiopian hospitals was found to be 72.2% (95% confidence interval: 59.1, 85.3%). Based on severity, the prevalence of major, moderate, and minor potential drug-drug interaction was 25.1, 52.8, 16.9%, respectively, also 1.27% for contraindications. The factors associated with potential drug-drug interactions were related to patient characteristics such as polypharmacy, age, comorbid disease, and hospital stay.

**Conclusions:**

There is a high prevalence of potential drug-drug interactions in Ethiopian hospitals. Polypharmacy, age, comorbid disease, and hospital stay were the risk factors associated with potential drug-drug interactions.

## Background

Drug-drug interactions (DDIs) are types of adverse drug events (ADEs) that can occur when the effect of a drug is altered by another drug that is taken. Commonly it ends up with a qualitative and/or quantitative change in drug action [1]. They may change the diagnostic, preventive, and therapeutic activity of any drug and results in treatment failure, the toxicity of medications, and alternation of drug efficacy [2].

It can be categorized based on the severity and mechanisms by which drugs interact with each other [3, 4]. Based on their severity, DDIs can be mild, moderate, or severe. Major DDIs may be life-threatening or may cause prolonged or permanent damage. Moderate DDIs may require medical intervention or change in therapy. Whereas minor DDIs do not usually require a change in therapy. Regardless of the DDI severity, the patient should be monitored for possible manifestations of the interaction [3]. DDIs can also be classified as pharmaceutical, pharmacokinetic, and pharmacodynamics based on the mechanisms of how drugs interact with each other [2].

There are different factors for the occurrence of potential DDIs. The age of the patient, common disease state and polypharmacy; pharmacokinetic and pharmacodynamic nature of drugs; the influence of disease on drug metabolism; prescriber issues such as multiple drug prescription by multiple prescribers, inadequate knowledge of prescribers’ on DDIs or poor recognition of the relevance of DDIs by prescribers are among the risk factors significantly associated with the occurrence of potential DDIs [5–10].

DDIs are common in cardiovascular, Human Immunodeficiency Virus-infected, psychiatric patients, and renal and hepatic insufficiency (CKD, cirrhosis) patients. Because this type of patient requires multiple types of drugs, their kidney and liver may decrease the excretion and metabolize the ability of medications. Therefore, the occurrence of DDIs in this type of patient may be significant [5–7, 11, 12].

DDIs are also more frequent in hospitalized patients, patients who stay in the hospital for a longer time, and/or receive more drugs per day [13–16]. Hospitalized patients are more likely to be affected by DDIs because of severe and multiple illnesses, comorbid conditions, chronic therapeutic regimens, poly-pharmacy, and frequent modification in therapy [17]. Among hospitalized patients, elderly patients are at higher risk of potential DDIs, and the occurrence of potential DDIs ranges from 3 to 69%, depending on the specific area and population. The increased prevalence was found to be related to the presence of multiple chronic illnesses, the use of multiple medications, and altered pharmacokinetics in elderly patients [8].

Physicians and pharmacists alert fatigue is a common reason for the occurrence of drug-drug interactions for patients receiving interacting drugs. Even though computerized DDI alert systems could decrease the occurrence of DDIs, numerous alerts produced by such system lead physician and pharmacist alert fatigue. This alert fatigue results in a considerable override of DDI alerts. A study done in Japan showed physicians overrode DDI alerts at a high rate in computerized drug interaction alert system [18].

DDIs may have undesirable or harmful effects in addition to their desirable effects [4]. Clinically significant DDIs may cause potential harm to patients, harmful outcomes, and resulting in an estimated cost of more than $1 billion per year to governmental health care system expenditure [19].

DDI is being an evolving public health problem currently [20]. In Ethiopia, now a day, polypharmacy is increasing due to a rise in the occurrence of comorbid conditions in the hospital health care system [21, 22], where large number of patients are hospitalized. So, there is a high possibility of DDIs. Furthermore, due to economic problems, the probability of monitoring patients with comorbid diseases using sophisticated instruments is not feasible; causing the patient to DDIs.

As a result, potential DDIs causing serious risk to patient health. Therefore, this study attempted to review and quantitatively estimate the prevalence of potential DDIs and associated risk factors in hospitals, both among inpatients and outpatients in Ethiopia.

## Methods

### Study protocol

The review protocol was created based on Preferred Reporting Items for Systematic Review and Meta-analysis (PRISMA). The checklist was strictly followed while reporting this systematic review and meta-analysis (Additional file 1: Table 1) [23]. The review protocol is registered on PROSPERO with reference ID number: CDR 42020149416. The published methodology is also available at https://www.crd.york.ac.uk/prospero/display_recored.php?ID=CDR42020149416.

### Screening and eligibility of studies

WA designed the study. Two authors WA and GA screened the title and abstracts of the studies based on the inclusion and exclusion criteria. They also collected the full texts, evaluated the eligibility of the studies for final inclusion, assessed the quality of the study, and analyzed the data. AI commented on the review and meta-analysis.

### Inclusion and exclusion criteria

#### Inclusion criteria

- √ Observational studies addressing the prevalence of potential DDIs and conducted in Ethiopia (prospective, retrospective and descriptive cross-sectional studies)
- √ All male and female patients in any age (pediatrics, adults, and geriatric) and admitted to hospital wards or visited outpatients
- √ All published articles without time limit
- √ Patients who had any disease and admitted to hospital wards or visited outpatients
- √ Studies which were published by English language and provided sufficient data for the review

#### Exclusion criteria

- √ Articles with missing or insufficient outcomes
- √ Studies that were conducted in primary health care settings
- √ Articles not published in peer reviewed journal.

### Search strategy and data sources

We had searched literatures from a legitimate database such as HINARI, Science direct, PubMed/MEDLINE, Google Scholar, and Research Gate for English-language publications. The literature search was performed to retrieve relevant findings closely related to the prevalence of potential DDIs and associated factors with DDIs among outpatients and inpatients in Ethiopian hospitals.

The search was conducted with the aid of carefully selected search-words without specification in time. “Prevalence”, “occurrence”, “potential DDIs”, “associated factors” and “Ethiopia” were the search words used in this review and meta-analysis. AND/OR words were used for the identification of the articles. The search was conducted from August 3–25, 2019 and all published articles available online until the day of data collection were considered.

### Data extraction

A standardized data extraction form was prepared in Microsoft Excel by the investigators. Important information which was related to study characteristics such as: Region, Study area, Author, Year of publication, study design, Pathology, Target population, Study setting, Interaction database, Number of patients, Number of patients with DDIs, and lists of medications that caused the interactions were extracted. Moreover, the outcome of interest (Prevalence of DDIs (%), Potential DDIs (major, moderate and minor) and associated factors of DDIs) were also extracted.

Fourteen studies were selected based on their abstract, inclusion, and exclusion criteria. Studies were searched, identified, and screened from different search engines that are published in the English language.

### Quality assessment

The quality of the selected studies was performed. All selected studies were reviewed according to twelve criteria adapted from a previous study [24]. these criteria’s were: objectives of the study, the definition of constitutes of a DDI, DDI categories, DDI categories defined, mention of DDI reference, data collection method described clearly, setting in which study was conducted described, study subjects described, sampling and calculation of sample size described, potential or actual DDIs assessed, measures in place to ensure that results are valid and limitations of the study listed. Each criterion is related to a quality assessment criterion with scores 0 or 1 and the total quality scores ranged from 0 to 12 (scores 0 to 6 = poor quality, 7 to 9 scores = moderate quality, 10 to 12 points = high quality) (Table 1).

**Table 1:** Quality assessment of included studies in the review

Studies	Total scores	Quality
Gunasekaran et al., 2016 [25]	**9**	Moderate
Behailu Terefe Tesfaye et al., 2017 [6]	**12**	High
Diksis et al., 2019 [5]	**12**	High
Chelkeba L et al., 2013 [26]	**12**	High
B.Akshaya Srikanth et al., 2014 [27]	**12**	High
Admassie, et al., 2013 [28]	**10**	High
Henok Getachew et al., 2016 [29]	**12**	High
Teka et al., 2016 [30]	**12**	High
Zeru Gebretsadik et al., 2017 [31]	**11**	High
Haftay Berhane Mezgebe, 2015 [7]	**11**	High
Teklay et al., 2014 [32]	**11**	High
Yesuf TA, et al., 2017 [33]	**10**	High
Tesfaye and Nedi, 2017 [34]	**11**	High
Kibrom et al., 2018 [35]	**11**	High

### Outcome measurements

The outcome measure in this review and meta-analysis is the prevalence of potential DDIs. It primarily aimed to assess the pooled estimates of potential DDIs in the hospitals of Ethiopia. This study has also two secondary outcome measures: Associated risk factors for potential DDIs and number of potential DDIs (major, moderate, and minor) in Ethiopian hospitals.

### Data processing and statistical analysis

Analysis of the pooled estimate of outcome measures i.e. Prevalence of potential DDIs, as well as subgroup analysis, were done by Open Meta Analyst advanced software. CMA version-3 software was used for publication bias assessment. The presence of publication bias was evaluated by using Egger’s regression tests and presented with funnel plots of standard error. Furthermore, the precision was presented with the Logit event rate. A statistical test with a *P* value of less than 0.05 (one-tailed) was considered significant [36].

### Heterogeneity assessment

Heterogeneity may be defined as any type of variability between studies in a systematic review and meta-analysis. When there is variability in participants, interventions, and outcomes studied, we call it clinical heterogeneity. In this review and meta-analysis, Der Simonian and Laird’s random-effects model were used by considering clinical heterogeneity among studies. Variability in study design and risk of bias may be described as methodological heterogeneity [37].

Variation in intervention effects being evaluated in different studies is defined as statistical heterogeneity. This type of heterogeneity is usually a result of clinical or methodological heterogeneity or both among studies. Statistical heterogeneity is assessed by using Cochran’s Q- statistics, chi-squared and I^2^ tests. In this review and meta-analysis, clinical heterogeneity of studies was assessed using I^2^ statistics. Based on the result of the statistical test, I^2^ statistics value of less than 25% were considered as low heterogeneity and I^2^ statistics value from 50 to 75% and I^2^ statistics value greater than 75% were considered as medium and high heterogeneity respectively [38].

## Results

### Article search results

**Fig. 1:**
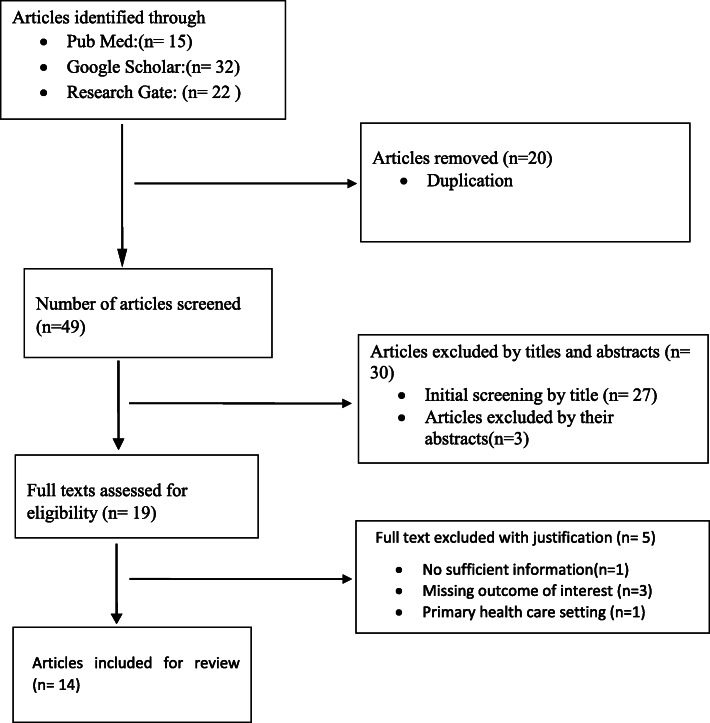
PRISMA flow diagram showing the selection process

A total of 69 articles were identified through the search strategy. After duplication was removed, 49 articles have remained for screening. From these, 30 articles were excluded by their titles and abstracts. The remaining 19 articles were then evaluated as per predetermined eligibility criteria for inclusion. Five articles were also excluded with justification (Additional file 2: Table 2). Finally, a total of 14 full-text articles that passed the eligibility criteria and quality assessment were included for final review and analysis (Fig. 1).

### General characteristics of the included studies

A total of 14 studies were included for systematic review and meta-analysis and important information that were related to study characteristics were presented in Table 2. All studies employed were observational cross-sectional study designs i.e. six retrospectives cross-sectional study (CS); three prospective CS and five CS design. The year of publication of included studies ranges from 2013 to 2019. The study included a wide range of population characteristics (pediatric, adult, and geriatric patients). Regarding geographic distribution, 14 studies were obtained from three regions and one city administration (Addis Ababa). The studies included all types of disease which had been treated in a medical ward and outpatient setting.

**Table 2:** General characteristics of studies included for systematic review and Meta-analysis

Region	Study area	Author and publication year	Study design	Pathology	Target population	Study setting	Interaction database
Oromia	Middle East Ethiopia, Adama	Gunasekaran et al., 2016 [25]	Retrospective CS	All	All hospitalized patients	All wards	Medscape online
	southeast of AA, Bishoftu	Behailu Terefe Tesfaye et al., 2017 [6]	CS	HIV/AIDS	All HIV infected patients	ART Clinic	Meds cape online & Drug.com
	South West Ethiopia, Jimma	Diksis et al., 2019 [5]	Prospective CS	Cardiac disorder	Cardiac adult patients	Medical ward	Micromedex 3.0 DRUG-REAX®
		Chelkeba L et al., 2013 [26]	CS	Cardiac disorder	Patients on CV medication in OPD	Cardiac clinic	Micromedex 2®
Amhara	North West Ethiopia, Gondar	B.Akshaya Srikanth et al., 2014 [27]	Prospective CS	All	All hospitalized patients	Medical ward	www.drugs.com
		Admassie, et al., 2013 [28]	Retrospective CS	All	All hospitalized patients	Inpatients and Out patients	Micromedex2®
		Henok Getachew et al., 2016 [29]	Retrospective CS	All	All hospitalized pediatric patients	Pediatric ward	Micromedex 2
Tigray	Northern Ethiopia	Teka et al., 2016 [30]	CS	All	All hospitalized elder patients	Medical ward	Micromedex® 2.0
		Zeru Gebretsadik et al., 2017 [31]	Retrospective CS	All	All patients who come for medical service	Outpatient pharmacy	Micromedex® 2.0
		Haftay Berhane Mezgebe, 2015 [7]	Retrospective CS	Psychiatric illness	Patients with psychiatric illness	Psychiatric unit	Micromedex 2.0 Drug-Reax®
		Teklay et al., 2014 [32]	Prospective CS	DVT	Patients on warfarin therapy	Medical ward	Micromedex® online
		Yesuf TA, et al., 2017 [33]	CS	All	All hospitalized patients	Medical ward	Micromedex 2®
AA	TASH	Tesfaye and Nedi, 2017 [34]	CS	All	All hospitalized patients	Medical ward	Medscape online
	SPHMMC	Kibrom et al., 2018 [35]	Retrospective CS	All	Adult patients	Medical ward	Micromedex 3.0 DRUG-REAX®

Abbreviations: *HIV* Human Immune Deficiency Virus, *AIDS* Acquire Immune Deficiency Syndrome, *ART* Antiretroviral Therapy, *CV* Cardio Vascular, *OPD* Outpatient Department, *CS* Crossectional Study, *TASH* Tikur Anbessa Specialized Hospital, *SPHMMC* Saint Paulos Millennium Medical College

Nine articles analyzed patients with all type of pathologies without focusing on any specific disease, two articles analyzed patients with the cardiac disorder, one article studied HIV patients and one article analyzed patients with psychiatric disorders.

Nine articles studied DDIs in inpatient ward (seven articles in a medical ward; one article in a pediatric ward; one article in all wards); four articles studied DDIs in the outpatient setting (ART Clinic, Cardiac Clinic, Psychiatric unit, and Outpatient pharmacy) and one article studied at inpatients and outpatient setting.

Among the fourteen studies analyzed, six different databases were used to detect potential interactions. About half of the studies used Micromedex® 2.0 database systems (seven articles; 50.0%), two articles (14.2%) used Medscape online, two articles (14.2%) used Micromedex® 3.0 database systems. The other three articles used Medscape online and drug.com, Drug.com and Micromedex online (Table 2).

### Quality of included studies

The quality of the included studies ranges from moderate to high quality (Additional file 3: Table 3).

### Study outcome measures

#### Prevalence of potential DDIs

**Fig. 2:**
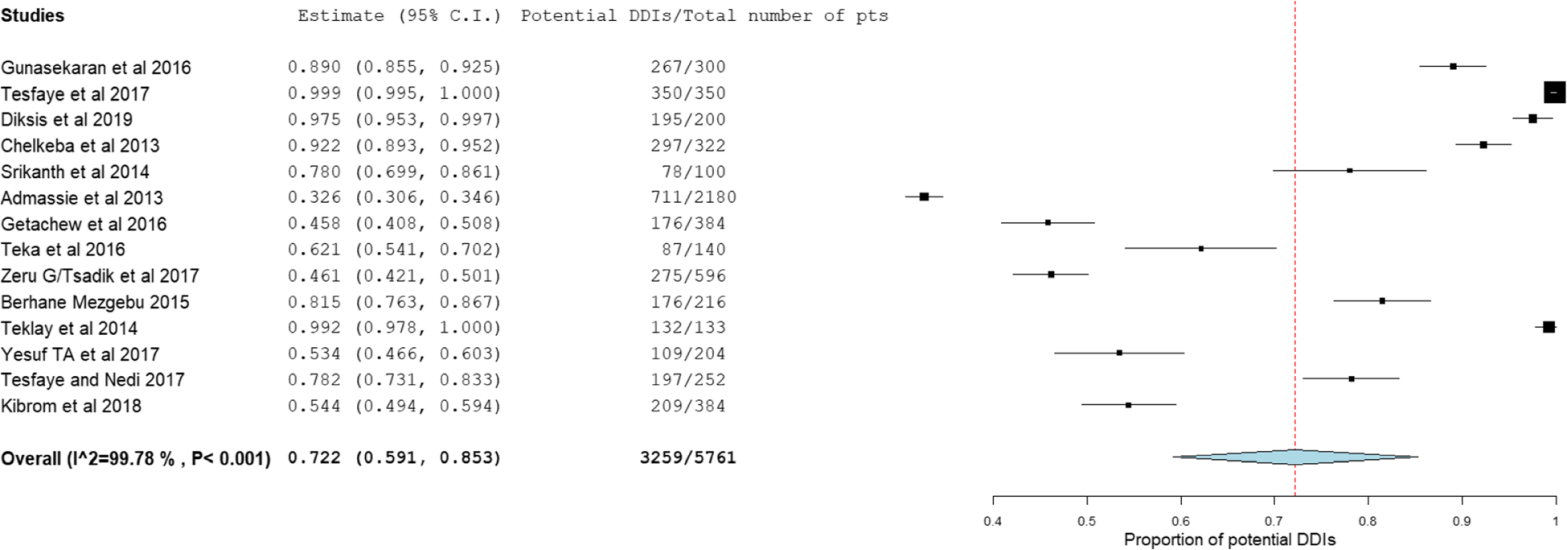
Forest plot depicting the pooled prevalence of patients with potential DDIs of 14 studies in Ethiopian Hospitals

**Fig. 3:**
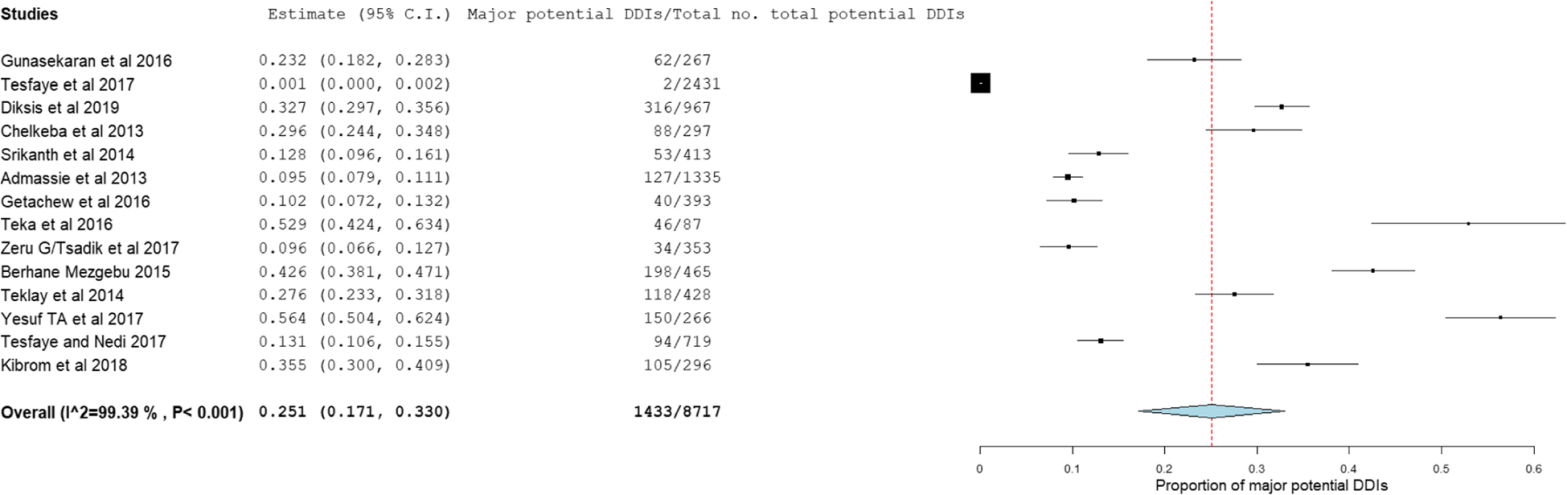
Forest plot depicting the pooled prevalence of major potential DDIs of 14 studies in Ethiopian Hospitals

**Fig. 4:**
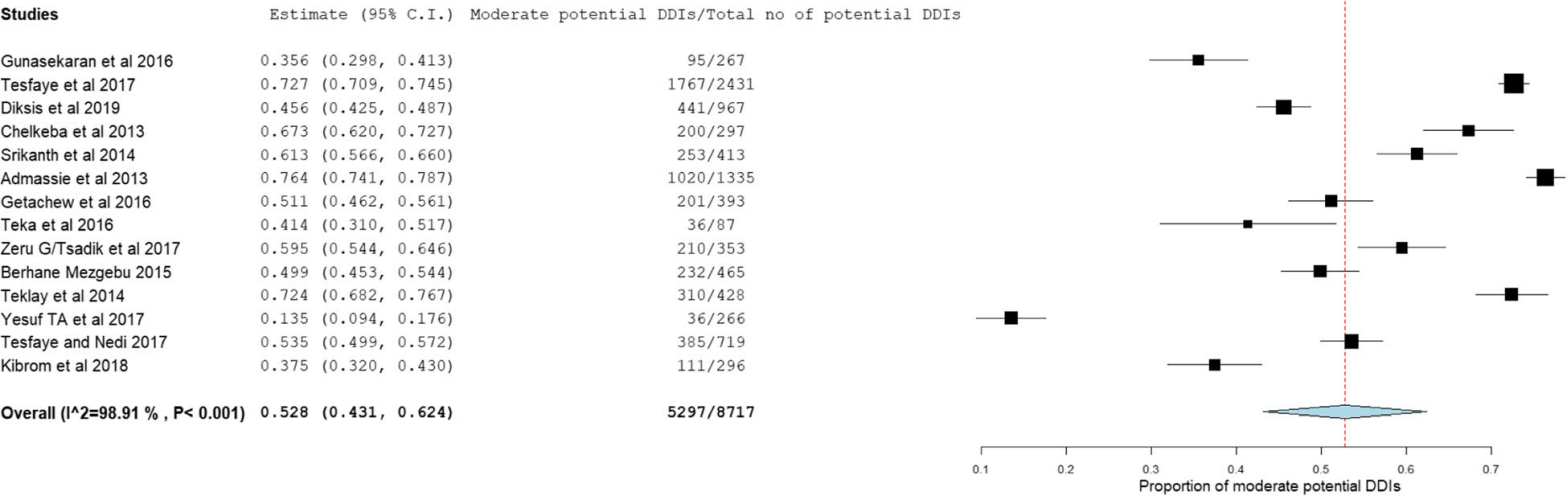
Forest plot depicting the pooled prevalence of moderate potential DDIs of 14 studies in Ethiopian Hospitals

**Fig. 5:**
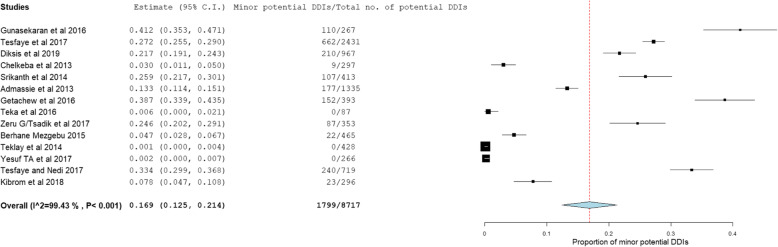
Forest plot depicting the pooled prevalence of minor potential DDIs of 14 studies in Ethiopian Hospitals

The prevalence and number of potential DDIs for each study are presented in Table 3. From 14 studies, the pooled prevalence of patients with potential DDIs in Ethiopian Hospitals was found to be 72.2% with 95% CI between 59.1 and 85.3). Figure 2 showed heterogeneity across 14 studies were high (I^2^ = 99.78%, *p* < 0.001). Based on the severity of DDIs, the pooled prevalence of potential DDIs was 25.1, 52.8, 16.9, and 1.27% for major, moderate, minor potential DDIs and contraindications respectively. Figures 3, 4, and 5 showed heterogeneity across 14 studies was high.

**Table 3:** Studies of the prevalence of potential DDIs in included articles

Region	Author	Pathology	Target population	Study setting	No. of patients	No. of patients with DDIs	Prevalence patients with DDIs (%)	No. of potential DDIs			
								Major	Moderate	Minor	Unknown& Contraindication
Oromia	Gunasekaran et al., 2016 [25]	All	All hospitalized patients	All wards	300	267	89.00	62 (23.2%)	95 (35.58%)	110 (41.2%)	
	Behailu Terefe Tesfaye et al., 2017 [6]	HIV/AIDS	All HIV infected patients	ART Clinic	350	350	100.00	2 (0.08%)	1767 (72.69%)	662 (27.2%)	
	Diksis et al., 2019 [5]	Cardiac disorder	Cardiac adult patients	Medical ward	200	195	97.50	316 (32.7%)	441 (45.6%)	210 (21.7%)	
	Chelkeba L et al., 2013 [26]	Cardiac disorder	Patients on CV medication in OPD	Cardiac clinic	322	297	92.24	88 (29.6%)	200 (67.34%)	9 (3.03%)	
Amhara	B.Akshaya Srikanth et al., 2014 [27]	All	All hospitalized patients	Medical ward	100	78	78.00	53 (12.8%)	253 (61.26%)	107 (25.9%)	
	Admassie, et al., 2013 [28]	All	All hospitalized patients	Inpatients and Out patient	2180	711	32.61	127 (9.59%)	1020 (77.04%)	177 (13.4%)	Contraindication = 11 (0.83%)
	Henok Getachew et al., 2016 [29]	All	All hospitalized pediatric patients	Pediatric ward	384	176	45.83	40 (10.2%)	201 (51.15%)	152 (38.7%)	
Tigray	Teka et al., 2016 [30]	All	All hospitalized elder patients	Medical ward	140	87	62.14	46 (51.6%)	36 (43.9%)	0 (0.0%)	Contraindication = 5 (6.1%)
	Zeru Gebretsadik et al., 2017 [31]	All	All patients who come for medical service	Outpatient pharmacy	596	275	46.14	34 (110.3%)	210 (63.444%)	87 (26.3%)	unknown = 22 (6.65%)
	Haftay Berhane Mezgebe, 2015 [7]	Psychiatric illness	Patients with psychiatric illness	Psychiatric unit	216	176	81.48	198 (43.8%)	232 (51.33%)	22 (4.87%)	Contraindication = 13 (2.88%)
	Teklay et al., 2014 [32]	DVT	Patients on warfarin therapy	Medical ward	133	132	99.25	11,827.6(%)	310 (72.43%)	0 (0.00%)	
	Yesuf TA, et al., 2017 [33]	All	All hospitalized patients	Medical ward	204	135	53.43	150 (80.6%)	36 (19.35%)	0 (0.00%)	Contraindication = 80 (43%)
Addis Ababa	Tesfaye and Nedi, 2017 [34]	All	All hospitalized patients	Medical ward	252	197	78.17	94 (13.1%)	385 (53.55%)	240 (33.4%)	
	Kibrom et al., 2018 [35]	All	Adult patients	Medical ward	384	209	54.43	105 (35.7%)	157 (53.4%)	32 (10.9%)	Contraindication = 2 (0.68%)

Abbreviations: *HIV* Human Immune Deficiency Virus, *AIDS* Acquire Immune Deficiency Syndrome, *ART* Antiretroviral Therapy, *CV* Cardio Vascular, *OPD* Outpatient Department

Based on the mechanisms of DDIs involved, seven studies documented well but the remaining seven studies didn’t document well the mechanisms of DDIs (Table 4).

**Table 4:** Studies of the prevalence of DDIs according to the mechanisms involved in Ethiopian Hospitals

Authors	Mechanism of DDIs		
	Pharmacokinetic	Pharmacodynamics	Unknown
Gunasekaran et al., 2016 [25]	164 (61.42%)	101 (37.83%)	2 (0.75%)
Behailu Terefe Tesfaye et al., 2017 [6]	1059 (43.56%)	1335 (54.92%)	37 (1.52%)
Diksis et al., 2019 [5]	245 (25.34%)	574 (59.36%)	148 (15.3%)
Henok Getachew et al., 2016 [29]	197 (50.13%)	181 (46.06%)	15 (3.82%)
Yesuf TA, et al., 2017 [33]	142 (53.38%)	124 (46.62%)	0 (0.0%)
Tesfaye and Nedi, 2017 [34]	358 (49.79%)	321 (44.65%)	40 (5.56%)
Kibrom et al., 2018 [35]	142 (47.97%)	87 (29.39%)	67 (22.6%)

Footnote: Seven studies did not report the mechanisms of drug-drug interaction

#### Factors associated with potential DDIs

The factors associated with potential DDIs were related to patient characteristics (Table 5).

**Table 5:** Associated factors for potential DDIs

Factors	Description
No of prescribed drugs (Polypharmacy)	Patients taking three or more than three concomitant drugs are at higher risk of the occurrence of potential DDIs [27, 28] There is an association of the occurrence of one or more potential DDIs with the number of medications prescribed per patient who took more than four medications [35] Polypharmacy (five or more medications) is an important factor which leads to potential DDIs [5, 29–31, 33, 34]
Co-morbid disease	Co-morbid condition independently increased the potential DDIs almost 2-folds [33]
Age	Older age was found to be predisposing factors for the occurrence of DDI [5, 28, 30, 31] Potential DDIs were occurring more frequently in the age group of 2–6 years than any other age group of the pediatric population [29]
Hospital stay	The chance of taking multiple drugs increases with longer stays (greater than or equal to seven) in the hospital, which in turn increases the risk for potential DDIs [5]
International Normalized ratio (INR value)	Increase in international normalized ratio value was found to be strongly associated with DDI and hence the risk of bleeding [32]

Footnote: Ten studies did not report the mechanisms of drug-drug interaction

#### Common interacting drug-combinations

The most common contraindications, major, and moderate DDIs are presented in Table 6.

**Table 6:** Most common contraindication, major and moderate DDIs identified in the included studies

Drug interaction pairs	Number of interactions	Severity	Effect of interaction
Clarithromycin+ simvastatin	6	Contraindication	Increased risk of myopathy or rhabdomyolysis
Chlorpromazine +Thioridazine	4	Contraindication	Risk of an irregular heartbeat which may belief threatening
Clarithromycin ciprofloxacin	1	Contraindication	Increased risk of QT interval prolongation
Aspirin+clopidogrel	160	Major	Bleeding
Aspirin+enalapril	157	Major	Renal dysfunction
Spironolactone + enalapril	101	Major	Hyperkalemia
Omeprazole+clopidogrel	56	Major	Decrease effect of clopidogrel and increased risk for thrombosis
Spironolactone + digoxin	47	Major	Increased risk of digoxin toxicity
Heparin + aspirin	38	Major	Increased risk of bleeding
Aspirin+furosemide	173	Moderate	Fluid retention
Haloperidol+Trihexphenidyl	74	Moderate	Decrease the effect of Trihexyphenidyl
Enalapril +Furosemide	59	Moderate	Postural hypotension (first dose)
Simvastatin+azithromycin	39	Moderate	Increased risk of rhabdomyolysis

### Test of heterogeneity, subgroup analysis, and publication bias

#### Test of heterogeneity

In this review and meta-analysis, there is clinical and statistical heterogeneity. The tests of heterogeneity showed significant heterogeneity (I^2^ = 99.78%, *p* < 0.001). To differentiate heterogeneity, sensitivity analysis, subgroup analysis, and Meta-regression was done.

#### Sensitivity analyses

There was no significant change in the degree of heterogeneity even if an attempt was done to exclude the expected outliers as well as one or more of the studies from the analysis. Therefore, fourteen studies were included for the meta-analysis.

#### Subgroup analyses

**Fig. 6:**
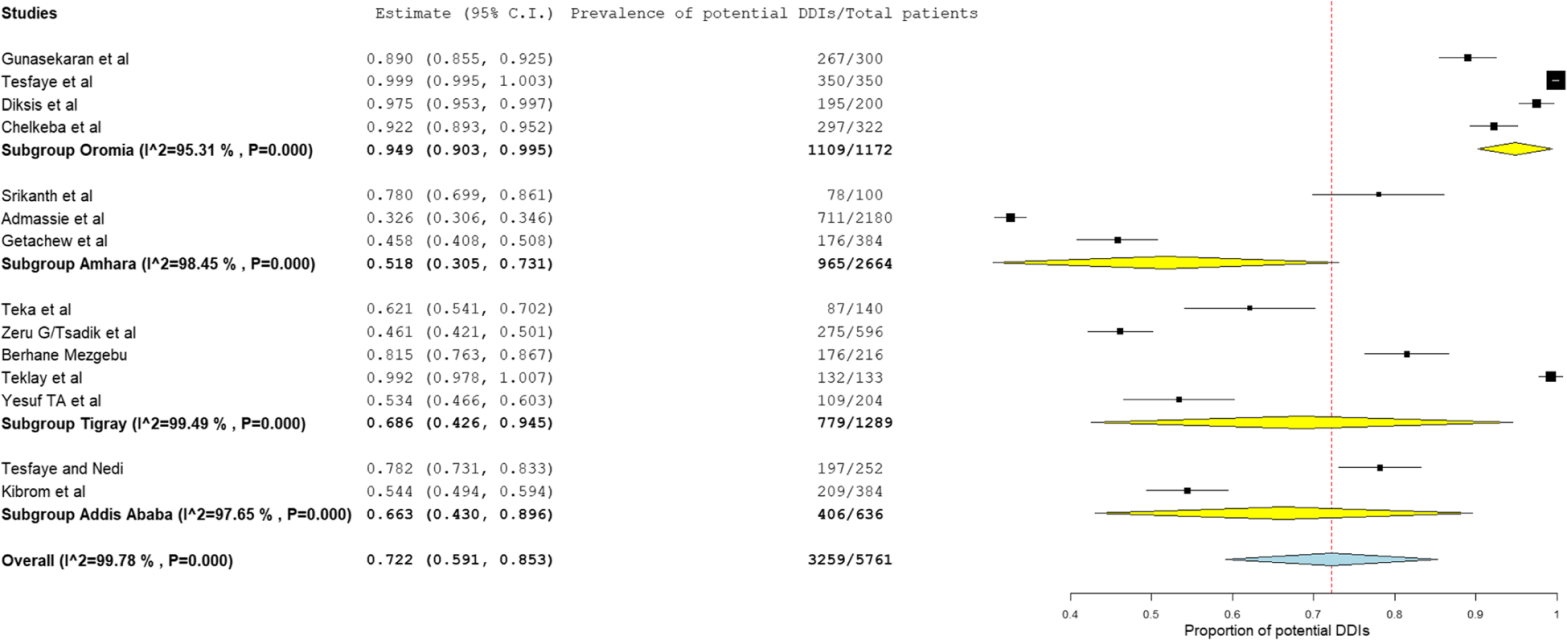
Subgroup analysis of the prevalence of potential DDIs based on region

Subgroup analysis also conducted based on Region and Study setting. Subgroup analysis based on a region revealed that the highest prevalence of potential DDIs was observed at Oromia Region, 94.9% (95% CI: 90.3 to 99.5) followed by Tigray Region with a prevalence of 68.6% (95% CI: 42.6 to 94.5) (Fig. 6).

Subgroup analysis based on study setting revealed that the highest prevalence of potential DDIs was observed at outpatient: 80.0% (95% CI: 58.9 to 101.1 followed by inpatient: 73.2% (95% CI: 60.8 to 85.7 and inpatient and outpatient setting: 32.6% (95% CI: 30.6 to 34.6).

**Fig. 7:**
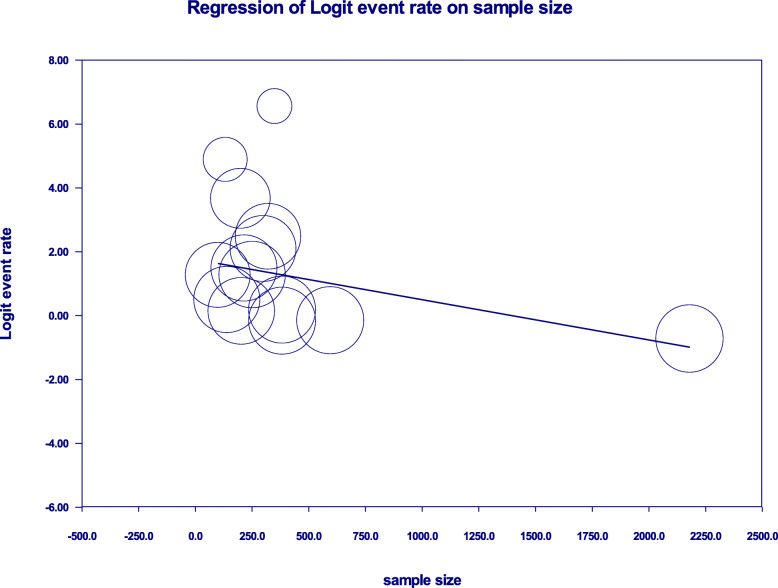
Univariate meta-regression model using sample size for the prevalence of potential DDIs

Univariate meta-regression for prevalence of potential DDIs revealed that sampling distribution is a source of heterogeneity (regression coefficient = 7.36; *p*-value = 0.0067) (Fig. 7).

### Publication bias

**Fig. 8:**
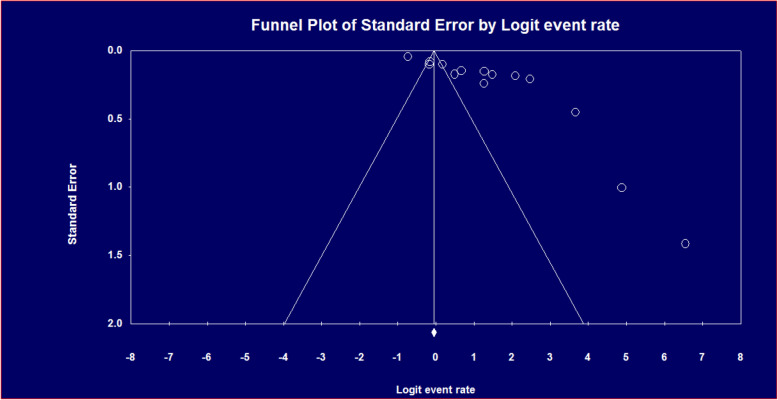
Publication bias using a funnel plot of standard error by Logit event rate

Funnel plots of standard error with logit effect size i.e. event rate supplemented by statistical tests confirmed that there is no evidence of publication bias on studies reporting the prevalence of potential DDIs and associated factors in Ethiopian Hospitals because there is no higher concentration of studies on one side of the mean than the other at the bottom of the plot (Fig. 8).

## Discussion

This systematic review and meta-analysis aimed to review and summarize the prevalence of potential DDIs and associated factors through reviewing and quantitatively summarizing the pieces of evidence available in Ethiopia. It was conducted and attempted to analyze 14 original studies addressing the topic. From all included studies, 5761 patients were included for pooled estimation of the primary outcome. A total of 8717 potential DDI was found in 3259 of patients. This means 2.67 DDIs per patient was suffering at least one DDI (calculated by dividing the number of potential DDIs/number of patients who suffer at least one potential DDI). On the other word, 1.5 DDIs were occurred per 100 patients (calculated by dividing the number of potential DDIs by the number of patients).

The overall prevalence of patients with potential DDIs in Ethiopia was found to be 72.2% (95%CI: 59.1, 85.3%). Based on the severity of DDIs, the pooled prevalence of potential DDIs was 25.1, 52.8, 16.9, and 1.27% for major, moderate, minor potential DDIs and contraindications respectively. These potential DDIs are more likely to produce negative outcomes. The analysis showed a high prevalence of DDIs which indicates the countries drug-drug interactions problem in the Ethiopians Hospitals. So, prescribers should prescribe interacting drugs in a monitored way.

The review showed that all DDIs studies in Ethiopia assessed potential DDIs, while no study was performed on actual DDIs. This may be due to identifying actual DDIs is much more complicated than potential DDIs.

The analysis showed that the occurrence of potential DDIs in the inpatient and outpatient settings reported by studies (inpatient: 73.2% (95% CI: 60.8 to 85.7%; outpatient: 80.0% (95% CI: 58.9 to 101.1%; inpatient and outpatient setting: 32.6% (95% CI: 30.6 to 34.6%). The prevalence of potential DDIs in this review is higher than another review in a developed nation in which 33% of the general population developed potential DDIs [39]. The high incidence of DDIs may be associated with a high number of drugs per prescription that was observed in individual studies. Otherwise, our review included only patients treated in the inpatient department, outpatient department, HIV clinic, and heart and cardiac clinics.

The prevalence of potential drug-drug interactions in the outpatient setting is higher than in the inpatient setting. The possible explanations for this finding. First, ART Clinic, Cardiac Clinic, Psychiatric unit, and Outpatient pharmacy were considered as outpatient settings. Moreover, the number of drugs and pathologies treated was different. This result helps hospitals to plan activities to prevent the occurrence of potential DDIs. So, hospitals can able to identify and follow up potential risk health care areas i.e. outpatient, inpatient, and other areas and help patients easily.

Similarly, this review showed all (100%) HIV infected patients treated in the outpatient setting [6]97.5% of adult patients with heart diseases treated in inpatient ward [5] and 92.23% cardiac disorder patients treated in the outpatient setting [26] were susceptible to DDIs. A high number of prescribed drugs, prescribing drugs with many potential DDIs, pharmacodynamics nature of drugs used in cardiology, and the influence of heart disease on drug metabolism may cause the high occurrence of potential DDIs in this group of patients. One finding in a developed country showed that 80% of hospitalized patients with heart diseases were susceptible to DDIs [40].

In this review and meta-analysis, age, polypharmacy, comorbid disease, and hospital stay were significantly associated with the occurrence of potential DDIs in the hospitals. Similarly, the finding from a review in a developed country highlighted these risk factors. Many studies had emphasized that the high occurrence of potential DDIs in old age is due to physiological changes related to age, comorbid diseases, and a high rate of medication use [41]. In addition to older age, potential DDIs were occurring more frequently in the age group of 2–6 years than any other age group of the pediatric population [29]. This is due to wide-ranging of patient ages and body-weights, limited physiologic reserve, medications dosing errors and ineptitude to properly communicate with healthcare workers [8].

Different studies were also supported as polypharmacy and comorbid disease increases the likelihood of the occurrence of potential DDIs [15, 33, 42, 43]. In the review, taking five or more medications was an important factor that leads to potential DDIs [5, 29–31, 33, 34]. This may be due to the probability of taking interacting drugs is increased. Likewise, the prevalence of potential DDIs from this review would likely have been higher.

Comorbid disease increases the occurrence of potential DDIs. Because the reason might be, the drugs prescribed for the comorbid disease are often used in combination that leads to the possibility of the occurrence of potential DDIs. Furthermore, increased hospital stay leads to the occurrence of potential DDIs. Since, hospitalized patients are more likely exposed to multiple illnesses, comorbid conditions, chronic therapeutic regimens, poly-pharmacy, and frequent modification during their stay of therapy [17].

The first limitation of this review and meta-analysis was the drug-drug interactions found were the only potential and doesn’t address the actual DDIs due to a lack of studies. Some of the studies included in the review and meta-analysis had small sample sizes. These might have led to bias. The other limitation of this review was Egger’s test funnel plots revealed as there is no publication bias but this estimation may not be accurate as small studies are included for the review and there are studies that had small size. The fourth limitation of this study was clinical heterogeneity among included studies, so it should be considered with caution. The classification of severity may be defined differently between studies, so this may be another limitation of this study.

## Conclusion

The prevalence of patients with potential DDIs in Ethiopian Hospitals was found to be high i.e. 72.2% (95% CI: 59.1, 85.3%). As of these, the most prevalent DDIs were moderate severity, 52.8%. In this review polypharmacy, age, comorbid disease, and hospital stay were the risk factors associated with potential DDIs. This review and meta-analysis had considerable clinical heterogeneity among included studies, so it should be considered with caution.

## Supplementary information

**Additional file 1: Table 1.**

**Additional file 2: Table 2.** Excluded studies after review of full text articles with justification.

**Additional file 3: Table 3.** Quality of included studies.

## Data Availability

All data generated or analyzed during this review are included in this published article.

## Abbreviations

ADEs: Adverse Drug Events
ART: Antiretroviral Therapy
CI: Confidence Interval
CMA: Comprehensive Meta-Analysis
CS: Cross-Sectional study
DDIs: Drug-Drug Interactions
PRISMA: Preferred Reporting Items for Systematic Review and Meta-Analysis
