# Supplementary material for: Prevalence of potential drug-drug interactions and associated factors among outpatients and inpatients in Ethiopian hospitals: a systematic review and meta-analysis of observational studies
Source: BMC Pharmacol Toxicol. 2020 Aug 24;21:63. doi: 10.1186/s40360-020-00441-2 (PMC7444065; doi:10.1186/s40360-020-00441-2)
Supplement: Supplementary file 2 — Additional file 2: Table 2. Excluded studies after review of full text articles with justification. [file 40360_2020_441_MOESM2_ESM.docx]

**Additional File 2: Table 2. Excluded studies after review of full text articles with justification**

| S.NO. | **Author** | **Year of publication** | **Title** | **Reason** |
| --- | --- | --- | --- | --- |
| 1 | A Anberber, D Berihun, W Shibeshi | 2015 | Prevalence and Characteristics of Polypharmacy and Drug-Drug Interaction in Afincho Ber Health Centre, Addis Ababa, Ethiopia | Primary health care setting |
| 2 | Teka F, Teklay G, Ayalew E, Kassa TT | 2016 | Prevalence of potentially inappropriate medications in ayder referral hospital, tigray region, northern Ethiopia: prospective study | Exclude by reading abstract |
| 3 | Alvetina Gall, Zerihun shenkute | 2009 | Ethiopian traditional and herbal medications and their interaction with conventional drugs | missing outcome of interest |
| 4 | Getachew Moges | 2013 | ASSESSMENT OF PHYSICIANS’ AWARENESS ON DRUGDRUG INTERACTIONS AND COMMON SOURCES OF  INFORMATION IN GENERAL HOSPITALS OF  ADDIS ABABA | missing outcome of interest |
| 5 | Tadesse TA, et al | 2018 | Prevalence of Warfarin Drug Interaction and Warfarin Education  Practice in Outpatient Setups of University Teaching Hospital: A  Retrospective Chart Review and an Observational Study | missing outcome of interest |
