# Supplementary material for: Prevalence of potential drug-drug interactions and associated factors among outpatients and inpatients in Ethiopian hospitals: a systematic review and meta-analysis of observational studies
Source: BMC Pharmacol Toxicol. 2020 Aug 24;21:63. doi: 10.1186/s40360-020-00441-2 (PMC7444065; doi:10.1186/s40360-020-00441-2)
Supplement: Supplementary file 3 — Additional file 3: Table 3. Quality of included studies. [file 40360_2020_441_MOESM3_ESM.docx]

| **Studies** | Objectives of the study clearly stated | Definition of what constitutes a DDI | DDI categories specified | DDI categories defined | Mention of DDI reference | Data collection method described clearly | Setting in which study was conducted described | Study subjects described | Sampling and calculation of sample size described | Potential or actual DDIs assessed | Measures in place to ensure that results are valid | Limitations of study listed | Total score | quality |
| --- | --- | --- | --- | --- | --- | --- | --- | --- | --- | --- | --- | --- | --- | --- |
| Gunasekaran et al | **1** | **1** | **1** | **0** | **1** | **1** | **1** | **1** | **0** | **1** | **0** | **1** | **9** | Moderate |
| Behailu Terefe Tesfaye et al | **1** | **1** | **1** | **1** | **1** | **1** | **1** | **1** | **1** | **1** | **1** | **1** | **12** | High |
| Diksis et al | **1** | **1** | **1** | **1** | **1** | **1** | **1** | **1** | **1** | **1** | **1** | **1** | **12** | High |
| Chelkeba L et al | **1** | **1** | **1** | **1** | **1** | **1** | **1** | **1** | **1** | **1** | **1** | **1** | **12** | High |
| B.Akshaya Srikanth et al. | **1** | **1** | **1** | **1** | **1** | **1** | **1** | **1** | **1** | **1** | **1** | **1** | **12** | High |
| Admassie, et al | **1** | **1** | **1** | **0** | **1** | **1** | **1** | **1** | **1** | **1** | **1** | **0** | **10** | High |
| Henok Getachew et al | **1** | **1** | **1** | **1** | **1** | **1** | **1** | **1** | **1** | **1** | **1** | **1** | **12** | High |
| Teka et al | **1** | **1** | **1** | **1** | **1** | **1** | **1** | **1** | **1** | **1** | **1** | **1** | **12** | High |
| Zeru Gebretsadik et al | **1** | **1** | **1** | **0** | **1** | **1** | **1** | **1** | **1** | **1** | **1** | **1** | **11** | High |
| Haftay Berhane Mezgebe | **1** | **1** | **1** | **1** | **1** | **1** | **1** | **1** | **1** | **1** | **1** | **0** | **11** | High |
| Teklay et al | **1** | **1** | **1** | **1** | **1** | **1** | **1** | **1** | **1** | **1** | **1** | **0** | **11** | High |
| Yesuf TA, et al | **1** | **1** | **1** | **0** | **1** |  | **1** | **1** | **1** | **1** | **1** | **0** | **10** | High |
| Tesfaye and Nedi | **1** | **1** | **1** | **1** | **1** | **1** | **1** | **1** | **0** | **1** | **1** | **1** | **11** | High |
| Kibrom et al | **1** | **1** | **1** | **1** | **1** | **1** | **1** | **1** | **1** | **1** | **1** | **0** | **11** | High |
